# Complete electromagnetic consideration of plasmon mode excitation in graphene rectangles by incident terahertz wave

**DOI:** 10.1038/s41598-024-58238-w

**Published:** 2024-03-30

**Authors:** K. V. Mashinsky, V. V. Popov, D. V. Fateev

**Affiliations:** 1 Kotelnikov Institute of Radio Engineering and Electronics of the Russian Academy of Sciences (Saratov Branch), Saratov, Russia 410019; 2 Saratov State University, Saratov, Russia 410012

**Keywords:** Graphene, Nanophotonics and plasmonics, Nanophotonics and plasmonics, Sub-wavelength optics, Terahertz optics

## Abstract

The excitation of terahertz plasmon modes in a graphene rectangle by normally incident linearly polarized electromagnetic wave has been theoretically studied. The complete electromagnetic approach based on formulation of the integral equations for sought-for electromagnetic quantities has been developed. The influence of edge-field effects on excitation of plasmon modes for different polarization of the incident wave and different shapes of graphene rectangle has been studied. The absorption cross-section spectra and the charge density distributions in graphene rectangle for different plasmon modes have been studied. It has been found that the edge-field effect, which results in spreading the plasmon field beyond the geometric boundaries of graphene rectangle, leads to considerable red shifts of the plasmon mode frequencies and modifies the plasmon mode dispersion.

## Introduction

Graphene plasmonics^1,2^ is a vibrant and rapidly developing area of flatland optoelectronics. Plasmons in graphene structures are able to localize the electromagnetic field down to a sub-wavelength scale that is two orders of magnitude shorter than the wavelength of electromagnetic radiation of the same frequency^3^.

Theoretical studies of terahertz (THz) plasmon excitations in two-dimensional electron systems (2DES) have been mainly conducted for infinitely wide 2DES or for 2DES which is confined only in one direction while infinite and homogeneous in the perpendicular direction^4–8^. Such a statement of the problem conveniently simplifies the solution of both the plasmon dispersion problem and problem of plasmon excitation by an external electromagnetic wave. This theoretical approach is applicable to 2DESs where the width of the structure greatly exceeds the confinement length of plasmons in longitudinal direction. In a realistic 2DES, all its planar dimensions are often comparable to each other and also comparable to the wavelength of plasmons in 2DES. In this case, the full electromagnetic analysis is needed to treat the problem of excitation of plasmon modes in 2DES cavity. The ultimately most symmetrical shape of 2DES cavity in the form of circular disk (and ring) has been studied^9–14^. Some theoretical works for studying the elliptical form of plasmonic cavity were performed^15,16^. Much fewer papers are devoted to the plasmon excitation in a rectangular 2DES (including graphene) cavity^17–19^. A rectangular 2DES cavity has a reduced symmetry as compared to disk (or ring) geometry and therefore the theoretical consideration becomes more complex. Theoretical approaches applied for studying the plasmon excitations in rectangular 2DES cavities employ either commercial numerical solvers^17,19,20^ or use simplifying approximations^18^.

A number of new plasmonic effects were found in spatially confined 2DES. It was shown that taking into account the electromagnetic retardation leads to lowering the resonant frequencies of plasmon excitations in 2DES stripe^21^. Plasmon propagation along 2DES strip was investigated both experimentally^22^ and theoretically^23^. Near-field microscopy was used for exciting and imaging plasmons in disk and rectangular graphene nanocavities and strong interaction between the edge^24,25^ and sheet plasmon modes was demonstrated^20^. Excitation of edge plasmons allows for stronger localization of THz field below the diffraction limit^26^. Lower-order plasmon modes in a square of 2DES in external magnetic field were studied both experimentally and theoretically in Ref.^18^. Plasmon resonances in graphene nanoribbons and nanoribbon arrays were investigated in Refs.^27–29^.

Commercial numerical solvers based on FEM or FDTD techniques for solving the electromagnetic (also plasmonic) problems in geometrically confined (even in all three dimensions) structures are often used^30–34^. Unfortunately, the FEM and FDTD techniques encounter serious difficulties when applied to problems where the electromagnetic processes of strongly different scales are involved. In particular, this happens when investigating the scattering of quite long THz electromagnetic wave by short-wavelength plasma oscillations in micro- and nanostructures. Therefore, other theoretical methods are also used in this case, such as the integral equation method^29^, plasmon wave eigen-function technique^30^, time-dependent density-functional^31^ etc.

In this paper, we solve the problem of plasmon excitation in graphene rectangular microcavity by an external electromagnetic (THz) wave in a self-consistent-field approach using the complete system of Maxwell equations. The integral equation method developed by us previously for 2DES confined only in one direction is extended for rectangular cavity geometry. Using this method, we investigate the properties of different plasmon modes excited by incident THz wave with linear polarization in graphene rectangle. It is shown that the edge-field effect in graphene rectangle plasmon cavity plays important role, leading to red shift of plasmon resonance frequencies and stronger dispersion of plasmon modes.

## Structure and methods

**Figure 1:**
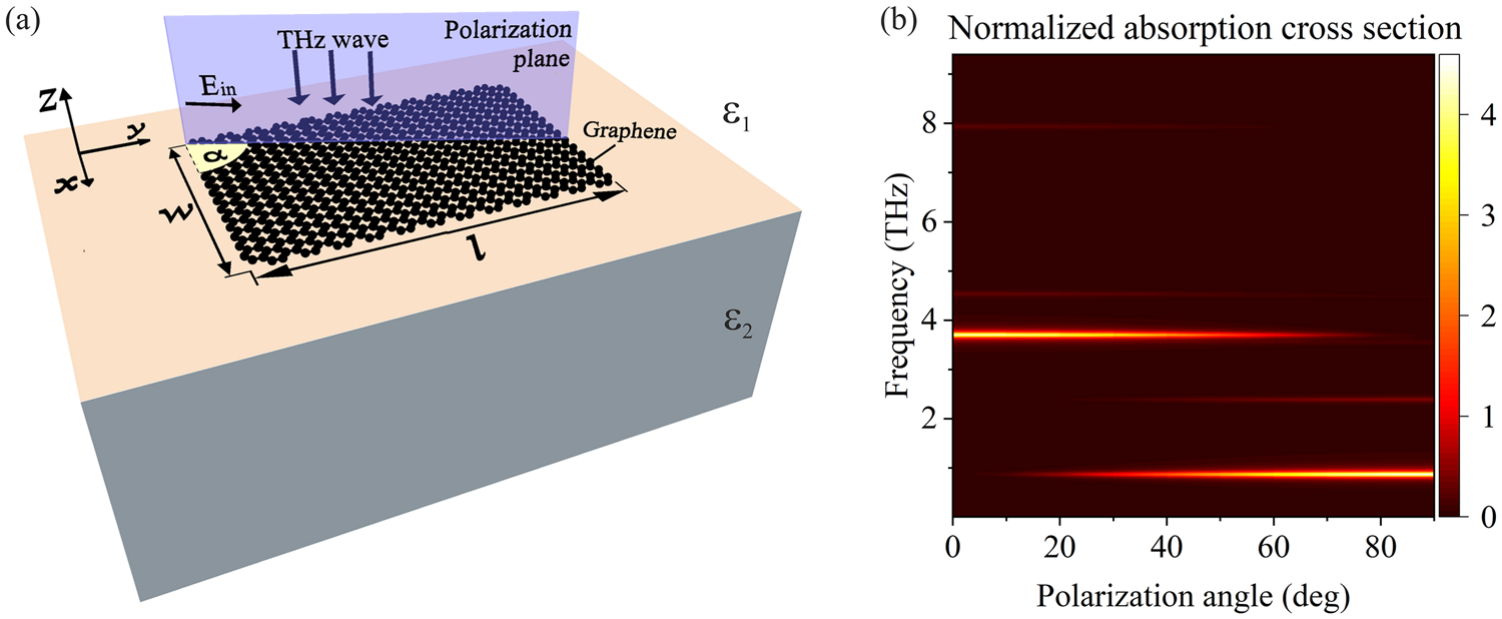
(**a**) Schematic representation of the studied graphene rectangle located on the interface between two semi-infinite media. (**b**) Dependence of the absorption cross-section (normalized to the geometric area of rectangle) on the frequency and polarization angle of the incident wave for *l* = 5 μm and *w* = 1 μm.

We study the excitation of plasmon modes in a graphene rectangle with sides *w* in the *x*-direction and *l* in the *y*-direction located in the *x*–*y* plane [Fig. 1(a)]. The *x–y* plane separates two half-spaces with dielectric constants *ε*_1_ and *ε*_2_. A linearly polarized electromagnetic wave is incident upon the *x–y* plane at normal direction from the medium with dielectric constant *ε*_1_.

The self-consistent-field electromagnetic method that we use consists of the following steps. At the first step, the electric and magnetic fields of the scattered waves are expressed by the double Fourier integral over the *x-* and *y-*coordinates, while the dependence on the *z*-coordinate is assumed to be exponential (only the expression for the electric field component *E*_*x*_ is given below as example):

$$E_{x}^{(1,2)} \left( {x,y,z,t} \right) = \exp \left( { - i\omega t} \right)\int\limits_{ - \infty }^{ + \infty } {\int\limits_{ - \infty }^{ + \infty } {E_{{x,q_{x} ,q_{y} }}^{(1,2)} \exp \left( {ik_{{z,q_{x} ,q_{y} }}^{(1,2)} z} \right)\exp \left( {iq_{y} y} \right)} \exp \left( {iq_{x} x} \right)dq_{x} } dq_{y} ,$$

where *ω* is the angular frequency, $E_{{x,q_{x} ,q_{y} }}^{(1,2)}$ are the spatial Fourier harmonics of the electric field $E_{x}^{{}} \left( {x,y,z,t} \right)$, superscripts 1 or 2 refer to the dielectric media with dielectric constants *ε*_1_ and *ε*_2_, respectively, $k_{{z,q_{x} ,q_{y} }}^{(1,2)}$ are the *z*-components of the wave vector in media 1 and 2, respectively, $q_{x}$ and $q_{y}$ are the in-plane components of the Fourier harmonic wave vector, which are the same in the both media in virtue of the boundary conditions in the *x–y* plane. Different components of the wave vector are related by the expression $k_{{z,q_{x} ,q_{y} }}^{(1,2)} = \sqrt {\frac{{\omega^{2} }}{{c^{2} }}\varepsilon_{1,2} - q_{x}^{2} - q_{y}^{2} }$.

We take into account that the scattered fields with the in-plane components of their wave vectors inside the light cone $\left( {q_{x}^{2} + q_{y}^{2} < \omega^{2} \varepsilon_{1,2} /c^{2} } \right)$ in a respective medium are outgoing from the structure waves with real component *k*_*z*_ (${\text{Re}} k_{{z,q_{x} ,q_{y} }}^{(1)} > 0$ and ${\text{Re}} k_{{z,q_{x} ,q_{y} }}^{(2)} < 0$) and the scattered fields for $\left( {q_{x}^{2} + q_{y}^{2} > \omega^{2} \varepsilon_{1,2} /c^{2} } \right)$ are the evanescent waves with imaginary *k*_*z*_ (${\text{Im}} k_{{z,q_{x} ,q_{y} }}^{(1)} > 0$ and ${\text{Im}} k_{{z,q_{x} ,q_{y} }}^{(2)} < 0$). In the second step, the Maxwell equations in the Fourier representation over the *x*- and *y*-coordinates are solved in semi-infinite dielectric media surrounding the graphene rectangle taking into account the boundary conditions for the in-plane components of the electric and magnetic fields in the interface between the dielectric semi-infinite media containing the graphene rectangle. We use the conventional boundary conditions for each Fourier-harmonic in the graphene plane *z* = 0, which are the continuity of the in-plane components of electric field and the discontinuity of the in-plane components of magnetic field with the jump defined by the current density in graphene when crossing the graphene plane* z* = 0 in *z*-direction. In the third step we formulate the system of integral equations for the components of electric currents densities $j_{x}$ and $j_{y}$ in graphene by using the obvious condition of zero electric current outside graphene rectangle in the plane *z* = 0 and Ohm’s law for electric current in graphene with the dynamic graphene conductivity $\sigma (\omega )$ given in Ref.^32^ with both the intraband and interband transitions of charge carriers included:

1 $$\begin{aligned} j_{x} (x,y) = & \sigma (\omega )\int\limits_{ - l/2}^{l/2} {\int\limits_{ - w/2}^{w/2} {j_{x} \left( {x^{\prime},y^{\prime}} \right)} } G_{{q_{x} ,q_{y} }}^{x,x} (x,x^{\prime},y,y^{\prime})dx^{\prime}dy^{\prime} \\ & \quad + \sigma (\omega )\int\limits_{ - l/2}^{l/2} {\int\limits_{ - w/2}^{w/2} {j_{y} \left( {x^{\prime},y^{\prime}} \right)G_{{q_{x} ,q_{y} }}^{x,y} (x,x^{\prime},y,y^{\prime})} } dx^{\prime}dy^{\prime} + \sigma (\omega )Z_{0x} E_{x}^{{\text{(in)}}} , \\ & \quad j_{y} (x,y) = \sigma (\omega )\int\limits_{ - l/2}^{l/2} {\int\limits_{ - w/2}^{w/2} {j_{x} \left( {x^{\prime},y^{\prime}} \right)} } G_{{q_{x} ,q_{y} }}^{y,x} (x,x^{\prime},y,y^{\prime})dx^{\prime}dy^{\prime} \\ & \quad + \sigma (\omega )\int\limits_{ - l/2}^{l/2} {\int\limits_{ - w/2}^{w/2} {j_{y} \left( {x^{\prime},y^{\prime}} \right)G_{{q_{x} ,q_{y} }}^{y,y} (x,x^{\prime},y,y^{\prime})} } dx^{\prime}dy^{\prime} + \sigma (\omega )Z_{0y} E_{y}^{{\text{(in)}}} , \\ \end{aligned}$$

where

$$G_{{q_{x} ,q_{y} }}^{m,k} (x,x^{\prime},y,y^{\prime}) = \frac{1}{{4\pi^{2} }}\int\limits_{ - \infty }^{ + \infty } {\int\limits_{ - \infty }^{ + \infty } {Z_{{m,k,q_{x} ,q_{y} }} j_{k} \left( {x^{\prime},y^{\prime}} \right)\exp \left( {iq_{x} \left( {x - x^{\prime}} \right)} \right)\exp \left( {iq_{y} \left( {y - y^{\prime}} \right)} \right)dq_{y} } dq_{x} }$$

are the kernels of the integral equations with superscripts *m* and *k* possessing the values *x* or *y*, and

$$\begin{gathered} Z_{{x,x,q_{x} ,q_{y} }} = - \frac{{\mu_{0} \omega }}{{k_{{z,q_{x} ,q_{y} }}^{(1)} - k_{{z,q_{x} ,q_{y} }}^{(2)} }} - \frac{{q_{x}^{2} }}{{\varepsilon_{0} \omega \left( {k_{{z,q_{x} ,q_{y} }}^{(2)} \varepsilon_{1} - k_{{z,q_{x} ,q_{y} }}^{(1)} \varepsilon_{2} } \right)}}, \hfill \\ Z_{{x,y,q_{x} ,q_{y} }} = Z_{{y,x,q_{x} ,q_{y} }} = - \frac{{q_{x} q_{y} }}{{\varepsilon_{0} \omega \left( {k_{{z,q_{x} ,q_{y} }}^{(2)} \varepsilon_{1} - k_{{z,q_{x} ,q_{y} }}^{(1)} \varepsilon_{2} } \right)}}, \hfill \\ Z_{{y,y,q_{x} ,q_{y} }} = - \frac{{\mu_{0} \omega }}{{k_{{z,q_{x} ,q_{y} }}^{(1)} - k_{{z,q_{x} ,q_{y} }}^{(2)} }} - \frac{{q_{y}^{2} }}{{\varepsilon_{0} \omega \left( {k_{{z,q_{x} ,q_{y} }}^{(2)} \varepsilon_{1} - k_{{z,q_{x} ,q_{y} }}^{(1)} \varepsilon_{2} } \right)}}, \hfill \\ \end{gathered}$$

$$Z_{0x} = Z_{0y} = \frac{{2\sqrt {\varepsilon_{1} } }}{{\sqrt {\varepsilon_{2} } + \sqrt {\varepsilon_{1} } }}$$

with *ε*_0_ and $\mu_{0}$ being the electric and magnetic constants. Finally, the system of integral equations is numerically solved by using the Galerkin procedure via expanding the sought-for current densities $j_{x}$ and $j_{y}$ in series by the Legendre polynomials over the *x-* and *y-*coordinates within the graphene rectangle:

$$\begin{gathered} j_{x} \left( {\chi_{x} ,\xi_{y} } \right) = \sum\limits_{{n,n_{1} = 0}}^{\infty } {\beta_{{n,n_{1} }}^{(x)} P_{n} \left( {\chi_{x} } \right)P_{{n_{1} }} \left( {\xi_{y} } \right)} , \hfill \\ j_{y} \left( {\chi_{x} ,\xi_{y} } \right) = \sum\limits_{{n,n_{1} = 0}}^{\infty } {\beta_{{n,n_{1} }}^{(y)} P_{n} \left( {\chi_{x} } \right)P_{{n_{1} }} \left( {\xi_{y} } \right),} \hfill \\ \end{gathered}$$

where $P_{n} \left( {\chi_{x} } \right)$ is the Legendre polynomial of the *n*-th degree, $\beta_{{n,n_{1} }}^{(m)}$ are the expansion coefficients, $\chi_{x} {\text{ and }}\xi_{y}$ are the normalized spatial coordinates reduced to the segment [-1,1] $(\chi_{x} = 2x/w, \, \xi_{y} = 2y/l).$ The Galerkin procedure transforms the system of integral equations into an infinite system of linear algebraic equations for the expansion coefficients $\beta_{{n,n_{1} }}^{(m)} .$ These expansions (and hence the system of linear algebraic equations) are truncated by taking into account only *N* polynomials over each coordinate to reach a desired level of the solution convergence. With retaining only *N* polynomials, the system of integral Eqs. (1) generates $2N^{2}$ linear algebraic equations for the expansion coefficients $\beta_{{n,n_{1} }}^{(m)} .$ The elements of matrix $\hat{M}$ of the system of linear algebraic equations $\hat{M}\hat{\beta } = \hat{R}$, where

$$\begin{gathered} \hat{M} = \left( {\begin{array}{*{20}c} {\hat{M}^{(x,x)} } & {\hat{M}^{(x,y)} } \\ {\hat{M}^{(y,x)} } & {\hat{M}^{(y,y)} } \\ \end{array} } \right), \hfill \\ \hat{\beta } = \left( {\begin{array}{*{20}c} {\hat{\beta }^{(x)} } \\ {\hat{\beta }^{(y)} } \\ \end{array} } \right), \hfill \\ \end{gathered}$$

and the column-vector of the free terms

$$\hat{R} = \left( {\begin{array}{*{20}c} {\hat{R}^{(x)} } \\ {\hat{R}^{(y)} } \\ \end{array} } \right),$$

are

$$\begin{gathered} M_{{\tilde{n},\tilde{r}}}^{(m,k)} = \sigma (\omega )\frac{lw}{{4\pi^{2} }}i^{{r_{1} + r}} i^{{n + n_{1} }} ( - 1)^{{n + n_{1} }} W_{{n_{1} ,r_{1} ,n,r}}^{(m,k)} - \delta_{m,k} \frac{{\delta_{n,r} }}{2r + 1}\frac{{\delta_{{n_{1} ,r_{1} }} }}{{2r_{1} + 1}}, \hfill \\ R_{{\tilde{r}}}^{(m)} = - \sigma (\omega )Z_{0m} E_{m}^{{\text{(in)}}} \delta_{{0,\tilde{r}}} , \hfill \\ \end{gathered}$$

where

$$W_{{n_{1} ,r_{1} ,n,r}}^{(m,k)} = \int\limits_{ - \infty }^{ + \infty } {\int\limits_{ - \infty }^{ + \infty } {Z_{{m,k,q_{x} ,q_{y} }} J_{{n_{1} }}^{{({\text{s}})}} \left( {\frac{{lq_{y} }}{2}} \right)J_{{r_{1} }}^{{({\text{s}})}} \left( {\frac{{lq_{y} }}{2}} \right)J_{n}^{{({\text{s}})}} \left( {\frac{{wq_{x} }}{2}} \right)} J_{r}^{{({\text{s}})}} \left( {\frac{{wq_{x} }}{2}} \right)} dq_{x} dq_{y}$$

with $J_{n}^{{({\text{s}})}} \left( {\frac{{wq_{x} }}{2}} \right)$ being the spherical Bessel function of the first kind of the *n*-th order, $\tilde{n} = nN + n_{1}$ and $\tilde{r} = rN + r_{1}$ are the matrix row and column numbers, respectively, $n$,$n_{1}$,$r$, and $r_{1}$ are the indexes of expanding the current densities $j_{x}$ and $j_{y}$ into series by the Legendre polynomials (each of them runs through all values from 0 to *N*–1), and $\delta_{i,j}$ is the Kronecker delta. The total size of the square matrix $\hat{M}$ is $2N^{2} \times 2N^{2} .$ The next problem is the numerical calculation of the matrix elements, each of which involves a double integral over wave vector components $q_{x}$ and $q_{y}$. The numerical integration of each integral $W_{{n_{1} ,r_{1} ,n,r}}^{(m,k)}$ involves separation the infinite integration interval into two subintervals. One subinterval within the light cone $q_{x} ,q_{y} \le \frac{\omega }{c}\sqrt \varepsilon$ and the other outside the light cone $q_{x} ,q_{y} > \frac{\omega }{c}\sqrt \varepsilon$ with $\varepsilon$ being the greatest dielectric constant of the media above and below graphene. This separation of the integration interval allows for speeding up calculations since, for sub-wavelength scatterers, the outgoing and evanescent scattered fields have strongly different wave vector scales. The resulting system of linear algebraic equations is solved using the Gaussian elimination method, by reducing the system matrix to a triangular form. To achieve the convergence within the error of 1% for describing the plasmon modes of our interest, we have to use typically *N* = 17 and integrate the spherical Bessel functions within the intervals $q_{x} < 400\frac{2\pi }{w}$ and $q_{y} < 400\frac{2\pi }{l}$.

Upon finding the current densities $j_{x}$ and $j_{y}$ in graphene rectangle, we can calculate the absorption cross section

2 $$\alpha_{{{\text{CS}}}} = \frac{A}{P},$$

where

3 $$A = {\text{Re}} \left( {\frac{1}{\sigma (\omega )}} \right)\int\limits_{ - w/2}^{w/2} {\int\limits_{ - l/2}^{l/2} {\left( {\left| {j_{x}^{{}} (x,y)} \right|^{2} + \left| {j_{y}^{{}} (x,y)} \right|^{2} } \right)dx} dy}$$

is the value of the absorption power and

$$P = \sqrt {\frac{{\varepsilon_{0} \varepsilon_{1} }}{{\mu_{0} }}} \left( {\left| {E_{in,x} } \right|^{2} + \left| {E_{in,y} } \right|^{2} } \right)$$

is the Pointing flux density of incident THz wave with *E*_in_ being the amplitude of the electric field of incident wave. In all figures below, we show the absorption cross-section normalized to the geometric area of graphene rectangle. The distribution of the charge density $\rho (x,y)$ in plasma oscillations over the graphene rectangle is calculated by the continuity equation

4 $$\rho (x,y) = - \frac{i}{\omega }\left( {\frac{{\partial j_{x} (x,y)}}{\partial x} + \frac{{\partial j_{y} (x,y)}}{\partial y}} \right).$$

In the calculations, we used the Fermi energy value ${\mathcal{E}}_{{\text{F}}} = 150 \, \;{\text{meV}}$, carrier momentum relaxation time $\tau = 2 \, \;{\text{ps}}$, and dielectric constants *ε*_1_ = 1 and *ε*_2_ = 4, which are typical parameters for graphene structures.

## Results and discussion

The dependence of the absorption cross section on frequency and polarization angle is shown in Fig. 1b for the length of graphene rectangle *l* = 5 μm and its width *w* = 1 μm. Generally, geometrical dimensions of graphene rectangle should be chosen being of the order of the plasmon wavelength in graphene (which is typically on the micron scale in THz frequency range). This allows for exciting the plasmon modes in graphene rectangle in THz frequencies^35^. Two types of absorption resonances are seen in this figure: the resonances predominantly excited by THz wave polarized at small angles *α* and resonances predominantly excited by THz wave polarized in the perpendicular direction. For an arbitrary polarization angle between 0 and 90°, both types of plasmon resonances are excited with different intensities. The frequencies of all absorption resonances are independent of the polarization angle. The strongest absorption occurs for the two fundamental (having the lowest frequencies) plasmon resonances excited for polarization angles near *α* = 0° and *α* = 90°. To simplify the consideration, we further consider two limiting cases of the incident wave polarization, *α* = 0° and *α* = 90°.

**Figure 2:**
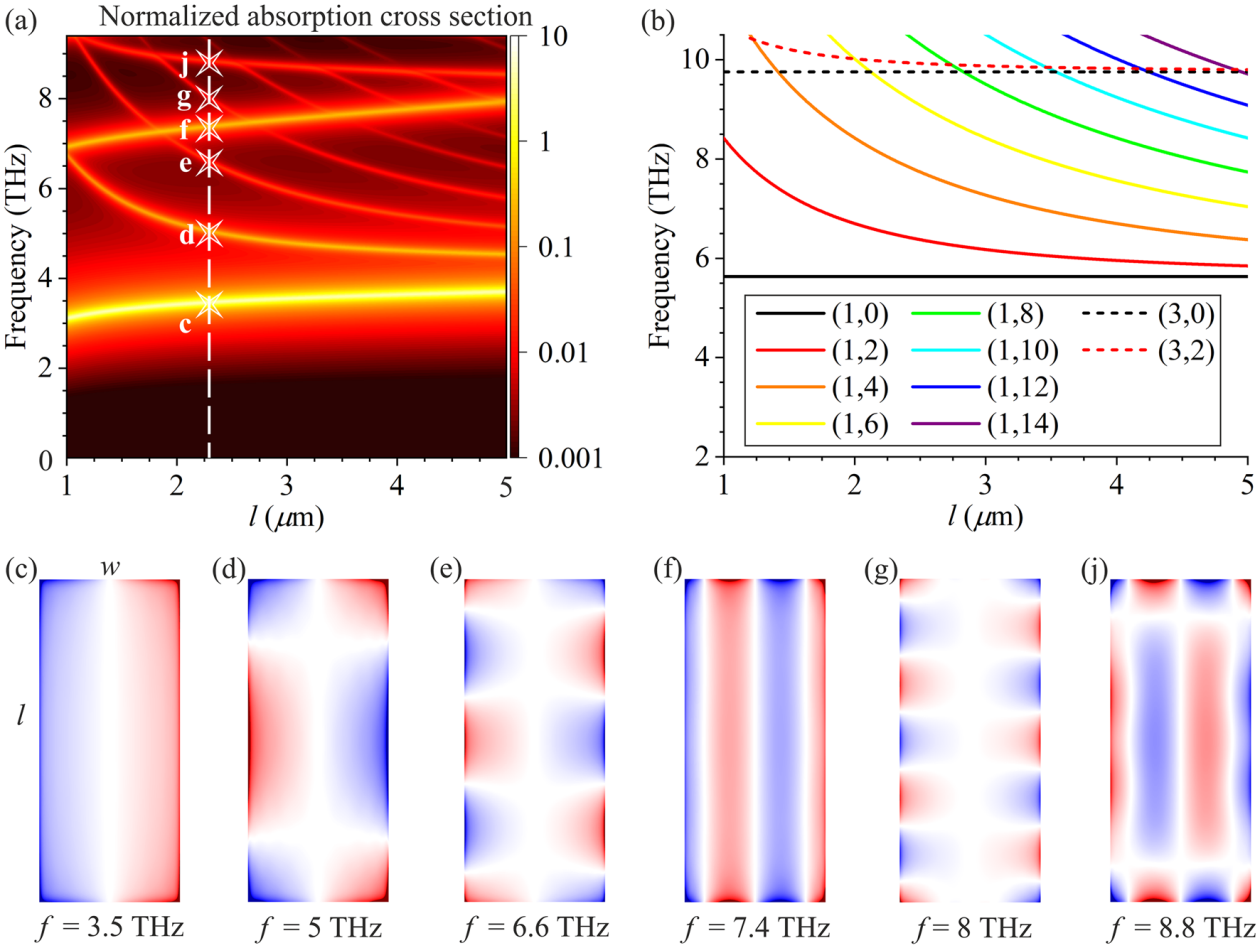
(**a**) The normalized absorption cross section for the *x*-polarized electric field in incident wave depending on frequency and the length of graphene rectangle *l* for *w* = 1 μm. Stars in the raster map in panel (a) denote the resonances for which the distributions of charge density oscillations are shown in panels (c–j). (**b**) Dispersion curves for different plasmon modes calculated by dispersion relation (5) as functions of frequency and the length of graphene rectangle *l* for *w* = 1 μm. (**c**–**j**) Distributions of charge density oscillations in graphene rectangle for *l* = 5 μm and *w* = 1 μm for different frequencies denoted by stars in panel (a). Blue and red colors in panels (c–j) correspond to the opposite phases of charge density oscillations.

Let us first consider the case when the electric field of incident wave is polarized along the shorter side *w* of graphene rectangle (in the *x*-direction). The normalized absorption cross section depending on the frequency of the incident THz wave and the length of the graphene rectangle *l* is shown in Fig. 2a. For better visualization of all resonances, the normalized absorption cross-section is shown in the logarithmic scale in Fig. 2a. We also show the instantaneous spatial distributions of the oscillating charge density $\rho$ in Fig. 2c–j for different plasmon resonances indicated by stars in Fig. 2a (for *l* = 2.25 μm). The charge density distributions for frequencies 3.5 THz [Fig. 2(c)] and 7.4 THz [Fig. 2(f)] correspond to the *bright* (i.e., strongly excited) plasmon modes with the charge density oscillations executing predominantly along the direction of electric field of the incident wave. The *bright* plasmon modes have strong dipole moments due to accumulation of charges of opposite signs at the opposite edges of graphene rectangle and hence these plasmon modes can be effectively excited by incident THz wave. The charge density in plasmon resonances for frequencies 5, 6.6, 8, and 8.8 THz shown in Fig. 2d,e,g,j oscillates in two perpendicular directions along different sides of graphene rectangle. Those are the resonances of *hybrid* plasmon modes in which the plasma oscillations of different parities (even and odd) in two mutually perpendicular *x*- and *y*-directions are involved. The plasma oscillations in the direction perpendicular to that of the electric field of incident wave arise due to confinement of the graphene rectangle in two perpendicular (*x*- and *y*-) directions. The charge density oscillations in all plasmon modes shown in Fig. 2d,e,g,j correspond to zero dipole moment of these oscillations in the *y*-direction and smaller dipole moment in the *x*-direction as compared to that for the *bright* plasmon modes shown in Fig. 2c,f. Therefore, the *hybrid* plasmon modes shown in Fig. 2d,e,g,j are darker (i.e., less excited) than the *bright* ones.

It is really instructive to compare the obtained results with the model of ideally reflecting boundaries for plasmons in graphene rectangle in the electrostatic approximation used in Ref.^18^.The ideal reflection boundaries model in the electrostatic approximation deals with the non-zero electric potential within the geometric area of graphene rectangle only and assumes it zero outside this area, while the complete electromagnetic approach that we employ considers the electric (and magnetic) fields in the whole space within and outside the geometric area of graphene rectangle self-consistently. Since the intraband transitions of charge carriers in graphene make a major contribution to the conductivity of graphene in THz frequencies, the contribution from the interband transitions can be neglected and the dispersion relation for plasmons in graphene (in the electrostatic approximation for lossless graphene) can be written as^36,37^

5 $$\omega = \frac{e}{\hbar }\sqrt {\frac{{{\mathcal{E}}_{{\text{F}}} }}{{\pi \varepsilon_{0} \left( {\varepsilon_{1} + \varepsilon_{2} } \right)}}|{\mathbf{q}}|} ,$$

where *e* is the elementary charge, **q** is the plasmon wave vector, and $\hbar$ is the reduced Planck constant. Expression (5) is valid for low temperatures $T \ll {\mathcal{E}}_{{\text{F}}} .$

In a rectangular plasmonic cavity with ideally reflecting boundaries, the selection rules for the *x*- and *y*-components of the plasmon wave vector are $q_{x} = {{p_{x} \pi } \mathord{\left/ {\vphantom {{p_{x} \pi } w}} \right. \kern-0pt} w}$ and ${{q_{y} = p_{y} \pi } \mathord{\left/ {\vphantom {{q_{y} = p_{y} \pi } l}} \right. \kern-0pt} l},$ where *p*_*x*_ and *p*_*y*_ are 0, 1, 2, 3 etc. We denote different plasmon modes by the indices $\left( {p_{x} ,p_{y} } \right)$ where index *p*_*x*_ indicates the number of charge density oscillation nodes in the graphene cavity in the *x*-direction while index *p*_*y*_ stands for the number of charge density oscillation nodes in the *y*-direction. In Fig. 2b, the dispersion curves for different plasmon modes calculated by dispersion relation (5) are plotted depending on the frequency and the length *l* of the graphene rectangle for *w* = 1 μm. Figure 2b shows the dispersion curves for two *bright* plasmon modes (1,0) and (3,0), and for a number of *hybrid* modes (those with even mode indices *p*_*y*_). The frequencies of the *bright* plasmon modes are independent of the graphene rectangle length *l* in the simplified model [see Fig. 2(b)]. The dispersion curve of any *hybrid* plasmon mode (*p*_*x*_,*p*_*y*_) merges into dispersion of the corresponding *brigh*t mode (*p*_*x*_,0) with increasing *l* because the wavelength of the *hybrid* mode with any finite mode index *p*_*y*_ along the *y*-direction grows to infinity as *l* tends to infinity. Therefore, the *hybrid* modes become indistinguishable from the *brigh*t mode (*p*_*x*_,0) in this case. Note that, in principle, the dispersion relation (5) describes also the plasmon modes with the even index *p*_*x*_ and both even indices *p*_*x*_ and *p*_*y*_. However, those plasmon modes cannot be excited by incident electromagnetic wave with the electric field polarized in the *x*-direction because they have zero dipole moments. Therefore, such plasmon modes are not seen in the absorption spectrum [Fig. 2(a)] and we do not show those modes in Fig. 2b.

The absorption cross section spectrum calculated in the electromagnetic approach [Fig. 2(a)] reveals substantial red shifts of the plasmon mode frequencies as compared with the plasmon mode dispersion in the simplified model [Fig. 2(b)]. This happens because, in the complete electromagnetic consideration, the fields of plasmon modes extend beyond the graphene rectangle boundaries so that the effective (electrical) area of plasmonic cavity exceeds its geometric area, while the latter is only taken into account in the simplified model with ideally reflecting boundaries of plasmonic cavity. The red shift of the plasmon mode frequency is much more pronounced for the fundamental plasmon mode (1,0). Its frequency in Fig. 2a is lower than that in Fig. 2b by a factor of 1.35 on the average for *l* between 1 and 5 μm. Decrease of the higher plasmon mode frequencies is smaller (but still pronounced) because the major part of higher plasmon mode oscillates within the geometric area of graphene rectangle. Also, the frequencies of the *bright* plasmon modes (1,0) and (3,0) change as a function of graphene rectangle length *l* in the complete electromagnetic consideration [Fig. 2(a)], while they are independent of *l* in the simplified model [Fig. 2(b)]. This is due to the edge-field effect seen at the lateral sides (with width *w*) of graphene rectangle in Fig. 2c,f. The role of edge-field effect becomes more pronounced for shorter *l* leading to even greater excess of the effective area of plasmonic cavity over its geometric area. The edge-field effect increases near the corners of graphene rectangle because of the strongest geometrical inhomogeneity in these areas.

**Figure 3:**
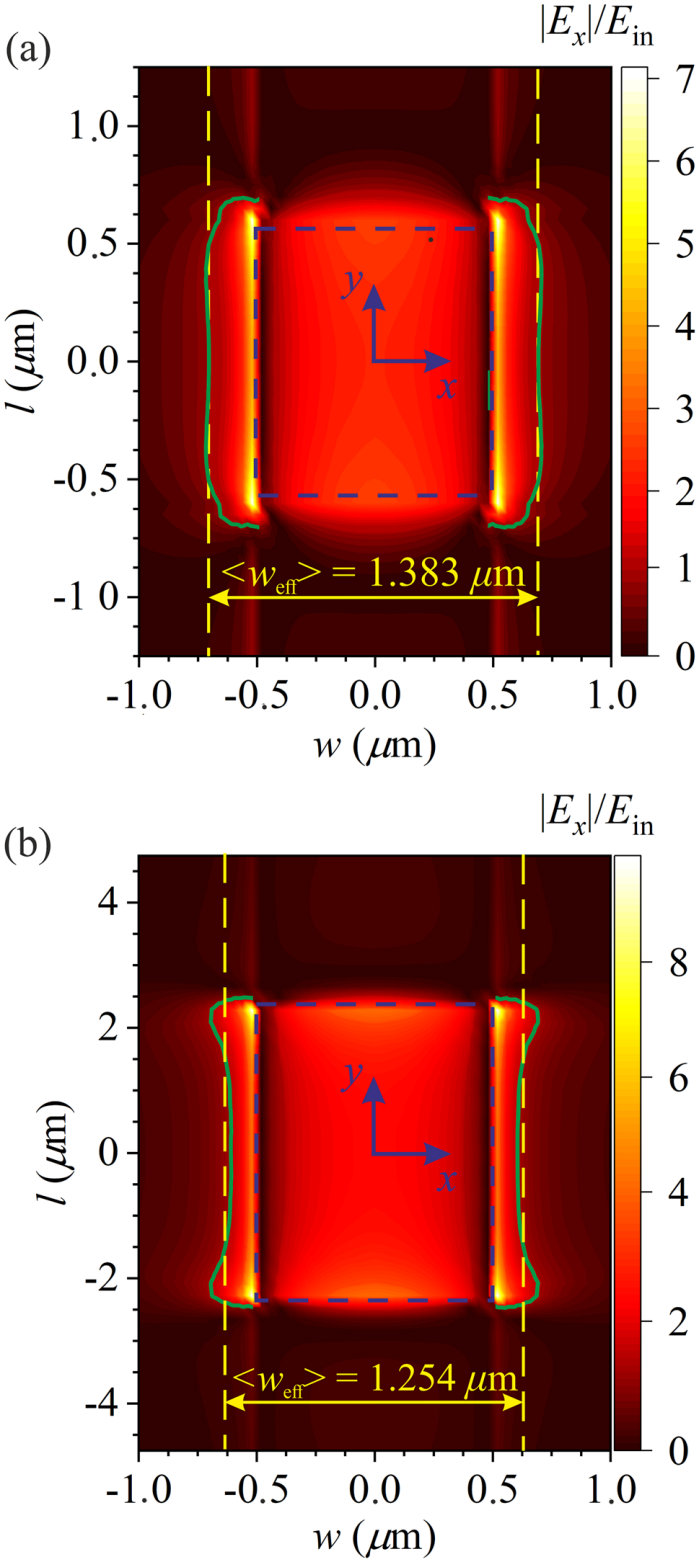
Distribution of the normalized amplitude of *E*_*x*_ component of electric field in the fundamental plasmon mode in the plane of a graphene rectangle for *w* = 1 μm and (**a**) *l* = 1.25 μm and (**b**) *l* = 4.75 μm. Blue dashed rectangles indicate the geometric boundaries of graphene. Yellow vertical straight dashed lines indicate the calculated average effective width $\left\langle {w_{{{\text{eff}}}} } \right\rangle$ of the plasmonic cavity. The plasmon field decreases by a factor of *e* as compared with the maximum electric field of the plasmon mode in the regions bounded by the green solid lines.

For the *bright* plasmon modes oscillating predominantly along the *x*-direction, the edge-field effect can be assessed in the simplest way by introducing the effective width *w*_eff_ of the plasmonic cavity along the direction of the electric field in incident wave. Effective width *w*_eff_ is defined as the width of the region covering the graphene rectangle in which the amplitude of the electric field component *E*_*x*_ decreases not more than by a factor of *e* (2.718) as compared with the maximum electric field amplitude of this plasmon mode in the cavity. Due to inhomogeneity of the edge-field effect, the effective width depends on the *y-*coordinate, and *w*_eff_(*y*) increases near the corners of graphene rectangle (see Fig. 3 for the fundamental plasmon mode). This effect can be integrally described by the average effective width of a graphene rectangle

6 $$\left\langle {w_{{{\text{eff}}}} } \right\rangle = \frac{1}{l}\int_{ - l/2}^{l/2} {w_{{{\text{eff}}}} (y)dy} ,$$

The electric field of the fundamental plasmon mode [shown in Fig. 3(a,b)] extends beyond the geometric boundaries of graphene rectangle with the average effective width of plasmonic cavity $\left\langle {w_{{{\text{eff}}}} } \right\rangle = 1.383w$ for *l* = 1.25 μm [Fig. 3(a)] and $\left\langle {w_{{{\text{eff}}}} } \right\rangle = 1.254w$ for *l* = 4.75 μm [Fig. 3(b)]. With decreasing *l*, the average effective width $\left\langle {w_{{{\text{eff}}}} } \right\rangle$ of the plasmonic cavity increases [cf. Figure 3(a) and 3(b)] and, therefore, the plasmon mode frequency decreases. This edge-effect leads to a decrease of the frequency of the fundamental plasmon mode by a factor of 1.13 with decreasing *l* from 5 to 1 μm [see Fig. 2(a)].

**Figure 4:**
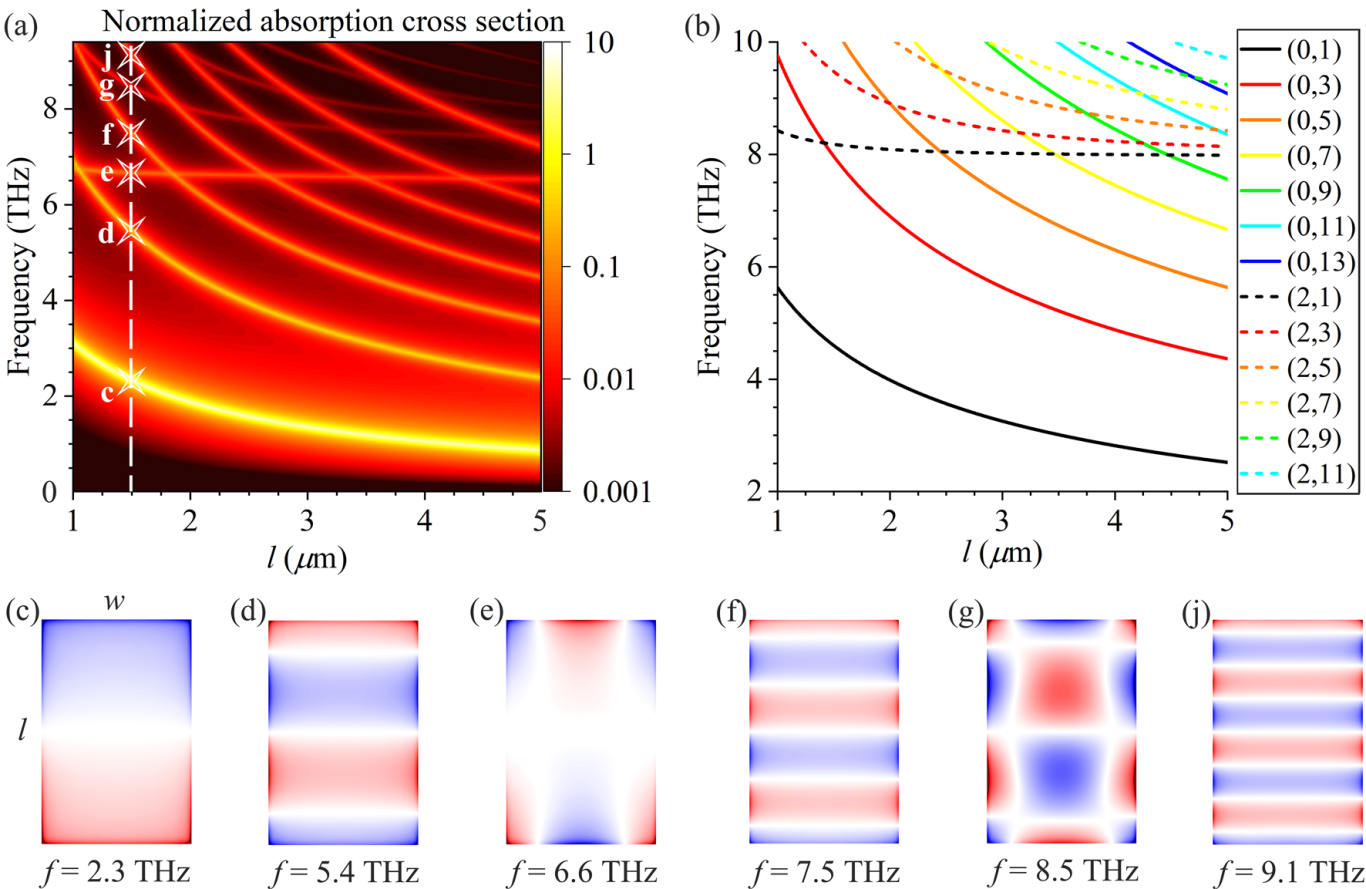
(**a**) The normalized absorption cross section as a function of frequency and the length of graphene rectangle *l* for polarization of the electric field of incident wave in the *x*-direction for the graphene rectangle width *w* = 1 μm. (**b**) Frequencies of different plasmon modes as functions of the length of graphene rectangle *l* calculated in the simplified model of ideally reflecting boundaries of graphene rectangle for *w* = 1 μm. (**c**–**j**) Charge density distributions of different plasmon modes in graphene rectangle for *w* = 1 μm and *l* = 1.5 μm at frequencies marked by stars in panel (a).

Now let us consider the polarization of electric field of the incident wave along the *y*-direction (*α* = 90°). The normalized absorption cross section calculated in the complete electromagnetic approach depending on frequency of the incident wave and the length of the graphene rectangle *l* is shown in Fig. 4a. The stars in Fig. 4a indicate the points for which the charge density distributions of different plasmon modes over the graphene rectangular cavity are plotted in Fig. 4c–j. For *α* = 90°, the *bright* plasmons oscillate predominantly along the *y*-direction [Fig. 4(c,d,f,j)]. Please note that the dispersion curves of any *hybrid* plasmon mode (*p*_*x*_,*p*_*y*_) merges asymptotically with increasing *l* into the dispersion curve of the *dark* (i.e., not exited) plasmon mode (*p*_*x*_,0) [not shown in Fig. 4(b)] which cannot be excited itself by incident THz wave. The reason is similar to that for *hybrid* plasmon modes (*p*_*x*_,*p*_*y*_) in Fig. 2 whose dispersion curves merge into dispersion curve of the corresponding (*brigh*t in that case) mode (*p*_*x*_,0) with increasing *l*. The wavelength of the *hybrid* mode with a finite even mode index *p*_*x*_ along the *y*-direction grows to infinity as *l* tends to infinity. Therefore, the *hybrid* modes become indistinguishable from the *dark* plasmon mode (*p*_*x*_,0) in this case. Similar to the case of polarization of the electric field of incident wave in the *x*-direction, we do not show the plasmon modes with the even index *p*_*y*_ and both even indices *p*_*x*_ and* p*_*y*_ because these modes cannot be excited by incident electromagnetic wave with the electric field polarized in the *y*-direction. Accordingly, such plasmon modes are not seen in the absorption spectrum in Fig. 4a.

As it was explained before, the *hybrid* modes are exited exclusively due to the edge-field effect at lateral sides of graphene rectangle. This effect is more pronounced for situation shown in Fig. 4 where the lateral width *w* of graphene rectangle is smaller than its longitudinal length *l*. Similarly to the case of *α* = 0 (Fig. 2), the frequencies of all plasmon modes shown in Fig. 4a calculated in the complete electromagnetic approach are red-shifted from those calculated in the simplified model, due to spreading the plasmon field beyond the geometric boundaries of graphene rectangle and, as a result, increasing the effective area of plasmonic cavity. For example, the frequencies of plasmon modes (0,1), (0,3), and (0,5) are red-shifted by factors of 1.6, 1.42, and 1.28, respectively, for *l* = 5 μm and *w* = 1 μm.

## Conclusion

The complete electromagnetic consideration of plasmon mode excitation in graphene rectangle by normally incident electromagnetic (THz) wave has been performed for different polarization of the incident wave. Polarizations of the electric field of incident wave along one or the other side of graphene rectangle are considered in detail. The properties of different types of plasmon modes have been studied for various aspect ratios of graphene rectangle. It was found that, when the electric field of incident wave is polarized along one of the sides of graphene rectangle, the *bright*-type plasmon modes executing predominantly along the electric field of incident THz wave are excited. The charge density distributions of these modes are odd across the area of graphene rectangle. The incident wave also excites the *hybrid* plasmon modes with even charge density distributions in the transverse direction. The complete electromagnetic analysis demonstrates that the plasmon field expands well beyond the geometric boundaries of graphene rectangle. This edge-field effect results in the increase of effective (electrical) plasmon cavity area and considerable red shifts of the frequencies of plasmon modes. The *hybrid* modes are exited exclusively due to the edge-field effect at lateral sides of graphene rectangle. Therefore, this effect is more pronounced for shorter lateral dimension of graphene rectangle. Our analysis reveals rather intricate character of the plasmon mode spectrum in a graphene cavity of rectangular shape. The results of this study can be useful for designing graphene devices with multimode plasmon cavities, such as THz detectors, mixers, sources, and near-field enhancement units for the THz near-field microscopy technique.

## Data Availability

The datasets generated during the current study are available from the corresponding author on reasonable request.
